# The relationship between the nursing practice environment and five nursing‐sensitive patient outcomes in acute care hospitals: A systematic review

**DOI:** 10.1002/nop2.828

**Published:** 2021-03-04

**Authors:** Tamer Al‐ghraiybah, Jenny Sim, Luise Lago

**Affiliations:** ^1^ School of Nursing & Affiliated Member of Illawarra Health & Medical Research Institute (IHMRI) University of Wollongong Wollongong NSW Australia; ^2^ Centre for Health Research Illawarra Shoalhaven Population Innovation Campus University of Wollongong Wollongong NSW Australia

**Keywords:** hospital‐acquired infection, medication administration error, mortality, nursing‐sensitive patient outcome, nursing practice environment, patient fall, PES‐NWI, pressure injury, pressure ulcer

## Abstract

**Aim:**

To synthesize the available evidence on the relationship between the nursing practice environment in acute care hospitals and five selected nursing‐sensitive patient outcomes (mortality, medication error, pressure injury, hospital‐acquired infection and patient fall).

**Design:**

A quantitative systematic review of literature was conducted using the PRISMA reporting guidelines (PROSPERO: CRD42020143104).

**Methods:**

A systematic review was undertaken up to October 2020 using: CINAHL, MEDLINE and Scopus. The review included studies exploring the relationship between the nursing practice environment in adult acute care settings and one of five selected patient outcomes using administrative data sources. Studies were published in English since 2000.

**Results:**

Ten studies were included. Seven studies reported that a favourable nursing practice environment reduced the likelihood of mortality in acute care hospitals, but estimates of the effect size varied. Evidence on the association between the nursing practice environment and medication administration error, pressure injury and hospital‐acquired infection was mixed.

## 1 INTRODUCTION

The last three decades have seen growing international interest in measuring the nursing practice environment (NPE). The NPE is defined as the “organizational characteristics of the work setting that facilitate or constrain professional nursing practice” (Lake, 2002, p. 178). Understanding the NPE will help nurse managers in acute care hospitals to improve the organizational characteristics that enhance or impede nurses to provide higher quality patient care (Duffield et al., 2011). These characteristics include four fundamental components: organizational management practices, workforce deployment practices, work design and organizational culture (Institute of Medicine, 2004). A small number of validated instruments measure the NPE with the most frequently used instrument being the Practice Environment Scale of the Nursing Work Index (PES‐NWI; Lake et al., 2019). There is established evidence linking the NPE with nurses' burnout (Lake et al., 2019), patient satisfaction (Bae, 2011) and some nursing‐sensitive patient outcomes (Lee & Scott, 2018; Stalpers et al., 2015).

Nursing‐sensitive patient outcomes are outcomes that are relevant to nurses' scope of practice where evidence has linked nursing inputs or interventions with patient outcomes (Sim et al., 2018; Stalpers et al., 2015). Nurses are not wholly responsible for these patient outcomes but they have a significant influence on the prevention of these outcomes occurring in hospitalized patients (Sim et al., 2018). Patient mortality, medication error, pressure injury, hospital‐acquired infection and patient fall are the most frequently examined patient outcomes (Doran, 2011; Dubois et al., 2017). The most commonly examined of these is mortality, which has been studied in relation to the NPE in a large number of primary studies (Lee & Scott, 2018). Only a small number of studies examine the association between the NPE and medication errors, pressure injuries, hospital‐acquired infections and patient falls. Research on the relationship between the NPE and these patient outcomes can provide nurses with actionable evidence to advocate for improvements in the NPE and potentially mitigate the risk of these preventable adverse events.

## 2 BACKGROUND

A variety of data sources and methods have been historically used to examine patient outcomes, which impacts on the ability to pool results and understand causation (Stalpers et al., 2015). Nurses' perceptions of frequency and/or severity of adverse events are commonly used to examine patient outcomes (Lee & Scott, 2018). Some studies use administrative data such as hospital discharge datasets, risk management or incident reporting systems or annual reports of clinical indicators (Sim et al., 2019; Stalpers et al., 2015). The Nursing Outcomes Classification (Moorhead et al., 2018) and standardized nursing languages are also being increasingly used to document nursing diagnoses, interventions and outcomes within electronic medical records (Oreofe & Oyenike, 2018). This heterogeneity in data sources leads to inconsistencies in measurement and is a challenge for synthesizing research findings.

The existing systematic reviews examining the NPE and patient outcomes combine data from nurse‐perceptions of the frequency of adverse events and administrative data sets (Bae, 2011; Lake et al., 2019; Lee & Scott, 2018). Only one systematic review included studies collecting patient outcomes (delirium, malnutrition, pain, patient falls and pressure injuries) from administrative data sources and they did not consider all of the NPE attributes (Stalpers et al., 2015). Most reviews examine the association between patient outcomes and nursing characteristics, such as nurse staffing, skill mix, nurse education levels, nurse satisfaction, nurse burnout and intention to leave (Bae, 2011; Lake et al., 2019; Lee & Scott, 2018; Stalpers et al., 2015). Only two reviews have investigated the link between the NPE and patient outcomes, and their findings were not conclusive (Bae, 2011; Lee & Scott, 2018). This review, therefore aims to: (a) review and synthesize the available evidence from administrative datasets on the relationship between the nursing practice environment and five selected nursing‐sensitive patient outcomes (mortality, medication error, pressure injury, hospital‐acquired infection and patient fall) and (b) describe how the nursing practice environment influences theses five patient outcomes.

### 2.1 Research question

What is the influence of the nursing practice environment on five nursing‐sensitive patient outcomes (mortality, medication error, pressure injury, hospital‐acquired infection and patient fall) among adults in acute care hospitals using administrative data sources?

## 3 THE STUDY

### 3.1 Design

This systematic review was conducted using the guidelines and procedures outlined in the Preferred Reporting Items for Systematic Review and Meta‐Analysis reporting guidelines (PRISMA; Moher et al., 2015) (See File S1). The review protocol was registered (CRD42020143104) in the international prospective register of systematic reviews (PROSPERO) at the University of York Centre for Reviews and Dissemination on April 28, 2020.

### 3.2 Method

#### 3.2.1 Search methods

The initial search was carried out in June 2019 and updated in October 2020 using three online bibliographic databases; Cumulative Index to Nursing and Allied Health Literature (CINAHL) Plus with full text, MEDLINE with full text and Scopus. The following search terms were used for the main independent variable: “practice environment” OR “nurs* work environment” OR “PES‐NWI” OR “practice environment scale” OR “nurse work index”. A separate search was performed for each outcome as described in Table 1. The search strategy was reviewed by a health librarian to improve the efficacy of the search.

**TABLE 1:** Search terms for Outcome measures

Outcome	Search terms
Mortality	Mortality OR “failure to rescue”
Medication error	“medication error” OR “medication administer* error”
Pressure injury	“pressure inj*” OR “bed sore” OR “pressure ulcer” OR “decubitus ulcer”
Hospital‐acquired infection	“healthcare associated infection” OR “nosocomial infection” OR “hospital acquired infection” OR “central line associated infection” OR “ventilat* associated bloodstream infection” OR “surgical site infection” OR “catheter associated urinary tract infection”
Patient fall	“fall*” OR “patient fall*”

#### 3.2.2 Inclusion/exclusion criteria

Research studies written in English, and published since 2000, were included if: (a) the study population included nurses working in acute care hospitals where the NPE was measured using a validated instrument; (b) the outcomes examined include adults with one of the five patient outcomes being investigated (mortality, medication error, pressure injury, hospital‐acquired infection and patient fall); and (c) data on the outcome of interest were empirically obtained from administrative data sources.

#### 3.2.3 Search outcomes

**FIGURE 1:**
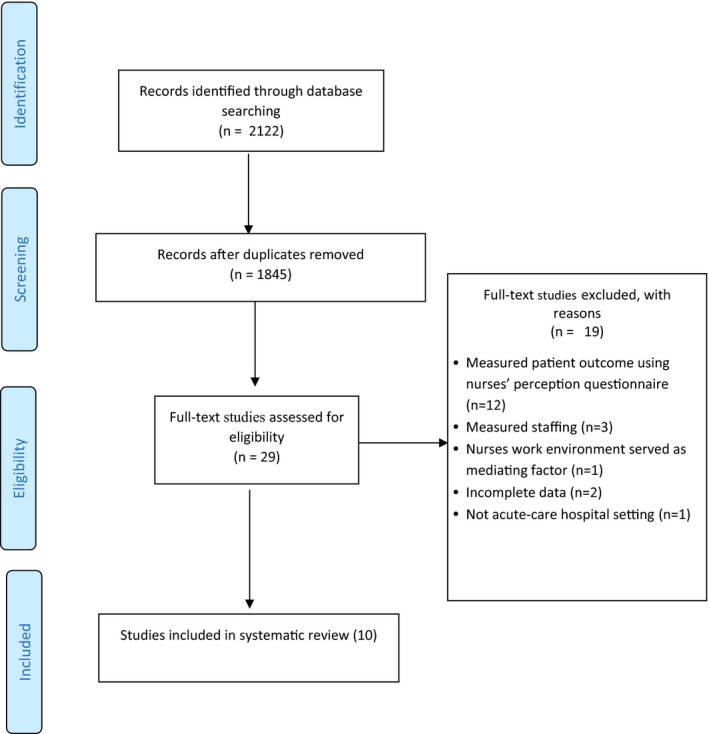
Process of paper selection – PRISMA Flow diagram (Moher et al., 2015)

A total of 2,122 studies were retrieved from online databases and stored using EndNote X9 (Clarivate Analytics, 2019). After removing duplicate titles, 1,845 studies were subject to title and abstract review by one researcher (TA). Twenty‐nine studies were subsequently subject to full‐text review which was undertaken independently by two researchers (TA and JS). Finally, 19 studies were excluded with reasons documented in Figure 1. A total of 10 studies were therefore included in the systematic review.

#### 3.2.4 Quality appraisal

The Appraisal tool for Cross‐Sectional Studies (AXIS; Downes et al., 2016) was used to assess the quality of all included studies. The AXIS tool includes 20 questions which are used to assess the research credibility and integrity of cross‐sectional studies in seven domains (Downes et al., 2016). Each question has three options: Yes, No or Unknown (Downes et al., 2016) and the appraisal process does not produce a numerical score.

Two researchers (TA and JS) independently appraised the methodological quality of the selected studies and then discussed and agreed on the final judgement of quality for each study. Ratings of good, moderate or poor were assigned to each study. Overall, all studies were of good quality but with common issues identified in two domains; six studies did not describe the process used for evaluating non‐responder bias and eight studies did not provide details about differences in characteristics between responders and non‐responders. There were no studies excluded based on quality appraisal. More information about the quality appraisal of all included studies is in File S2.

#### 3.2.5 Data extraction

Data were independently extracted by two researchers (TA and JS) into a summary table (Table 2). The extracted data included study characteristics (authors name, date of publication, location), study design, sample characteristics (sample size for nurses and patients) and measurement criteria (instrument used to assess the NPE). The association between the NPE and each patient outcome (mortality, medication error, pressure injury, hospital‐acquired infection and patient fall) was examined by extracting the regression coefficient, adjusted or unadjusted odds ratio (OR) and associated confidence intervals for each outcome in each study. If an odds ratio or regression coefficient was not reported, the correlation coefficient was extracted.

**TABLE 2:** Summary table of included studies

Author (year) Country	Aim	Study design	Sample	Scale	Outcomes	Analysis‐level	Statistical outcomes (95% confidence intervals)	Findings
Aiken et al. (2008) USA	To examine the effect of NPE on mortality	Cross‐sectional, retrospective	10,184 nurses and 232,342 patients from 168 hospitals	PES‐NWI 3‐subscales	Mortality	Hospital‐level	OR = 0.93 CI = (0.89–0.99) *p* < .05	The odds of patient mortality in hospitals with better NPE were lower by 7% than in hospitals with poor NPE
Aiken et al. (2011) USA	To assess the impact of NPE on patient outcomes	Cross‐Sectional retrospective	39,038 nurses and 1,262,120 patients from 665 hospitals	PES‐NWI 4‐subscales	Mortality	Hospital‐level	OR = 0.926 CI = (0.897–0.956) *p* < .0001	Each one‐standard deviation increase in the NPE mean score decreases the odds of patients dying, by 7% compared to hospitals with poor NPE
Cho et al. (2015) South Korea	To test the effect of staffing, NPE and education on mortality	Cross‐sectional retrospective	1,024 nurses and 76,036 patients from 14 hospitals (surgical units)	PES‐NWI 29‐items	Mortality	Hospital‐level	OR = 0.52 CI = (0.31–0.88) *p* = .01	The odds of patient mortality are 48% lower in hospitals with better NPE than in hospitals with mixed or poor NPE
Friese et al. (2008) USA	To examine the influence of NPE on mortality of hospitalized cancer patients undergoing surgery	Cross‐sectional retrospective	25,957 patients from 164 hospitals	PES‐NWI 31‐items	Mortality	Hospital‐level	OR = 1.30 CI = (1.01–1.68) *p* < .05	Patients in hospitals with unfavourable NPE had 30% increased odds of mortality compared to hospitals with mixed or good NPE
McHugh et al. (2016) USA	To examine the association between nurse staffing and NPE among patients who suffered in‐hospital cardiac arrests	Cross‐Sectional	11,160 patients from 75 hospitals	PES‐NWI 4‐subscales	Mortality	Hospital‐level	OR = 0.84 CI = (0.71–0.99) *p* < .05	Patient in hospitals with poor NPE had a 16% decreased odds of survival compared to hospitals with good or mixed NPE
Olds et al. (2017) USA	To determine whether NPE makes a distinct contribution to patient mortality	Cross‐sectional, retrospective	27,009 nurse and 852,974 patients from 600 hospitals	PES‐NWI 31‐items	Mortality	Hospital‐level	OR = 0.94 CI = (0.888–0.996) *p* = .035	An increase of one standard deviation in the mean NPE score decreased the odds of a patients dying by 6%
Fasolino and Snyder. (2012) USA	To examine the link between clinical contextual factors and medication administration errors	Cross‐sectional	248 nurse (11 medical‐surgical units)	PES‐NWI 29‐items	Medication error	Hospital‐level	*r* = .15 *p* < .01	The NPE was positively associated with medication error rates
Flynn et al. (2012) USA	To analyse the relationship among characteristics of the NPE, staffing, nurses' error practices interceptions, and rate of medication error	Cross‐sectional, retrospective	686 nurses from 14 hospitals (82 medical‐surgical units)	PES‐NWI 31‐items	Medication error	Unit‐level	β = −0.26 *SE* = 0.30 *p* = .388	There was no evidence of a relationship between NPE and medication error rate
Ma and Park (2015) USA	To explain how organizational nursing factors including NPE are associated with pressure injury	Cross‐sectional	33,845 nurses from 373 hospitals (1,381 units)	PES‐NWI 31‐items	Pressure Injury	Unit‐level	OR = 0.71 CI = (0.56–0.93) *p* = .013	Each one‐unit improvement in NPE score led to a reduced odds of developing a pressure injury by 29%
Stone el al. (2007) USA	To examine the effect of NPE on patient safety outcomes	Cross‐sectional	1,095 nurses and 15,846 patients from 31 hospitals (51 ICU)	Perceptions of Nurse Work Environment scale	CLBSI	Hospital‐level	OR = 1.19 CI = (1.05–1.36)	Each one‐unit improvement in Perceptions of Nurse Work Environment scale score was associated with 19% higher risk for developing a CLBSI
					CAUTI		OR = 0.61 CI = (0.44–0.83)	Each one‐unit improvement in Perceptions of Nurse Work Environment scale score was associated with a 39% reduction in the odds of developing a CAUTI
					VAP		OR = 1.03 CI = (0.79–1.34)	There was no evidence of an association between the NPE and developing VAP
					Pressure injury		OR = 1.06 CI = (0.83–1.37)	There was no evidence of an association between the NPE and developing a pressure injury
					Mortality		OR = 0.97 CI = (0.90–1.02)	There was no evidence of an association between the NPE and the odds of mortality

Abbreviations: CAUTI, Catheter‐associated urinary tract infection; CI, Confidence Interval; CLBSI, Central line‐associated blood stream infection; NPE, Nursing Practice Environment; OR, Odd Ratio; *p*, *p*‐value; PES‐NWI, Practice Environment Scale‐Nursing Work Index; *r*, spearman rank correlation; *SE*, Standard Error; VAP, Ventilator‐associated infection.

### 3.3 Synthesis

The synthesis without meta‐analysis (SWiM) guidelines for systematic reviews (Campbell et al., 2020) were used to guide data synthesis. The SWiM reporting guideline is intended to guide reporting of the synthesis of effect estimates when meta‐analysis is not used (Campbell et al., 2020). Meta‐analysis was not used in this review because of the large differences in the outcomes measures. Also, for each of the five patient outcomes, the population, study design and findings from included studies were analysed and synthesized. Measures of association between the NPE and each outcome, and statistical significance, were summarized. Finally, we described the limitations in the methodological approaches of included studies, and described how these could bias the results and limit generalizability.

## 4 RESULTS

### 4.1 Characteristics of included studies

Of the ten included studies, nine were conducted in the USA and one study was conducted in South Korea (Cho et al., 2015). No studies were undertaken in Australia, the United Kingdom or in Europe to assess the influence of the NPE on the selected patient outcomes using administrative data sources. No studies used the Nursing Outcomes Classification or standardized nursing languages. All included studies used a cross‐sectional design.

### 4.2 Nursing practice environment

Nine studies examined the NPE using the Practice Environment Scale‐Nursing Work Index (PES‐NWI) and one study used the 42‐item Perceptions of Nurse Work Environment scale (Stone et al., 2007). These two tools were derived from the same original instrument (Choi et al., 2004), the Nursing Work Index (Kramer & Laurin, 1989). The Perception of Nurse Work Environment Scale comprises four subscales; professional practice; nursing management; staffing and resources adequacy; and nursing process (Choi et al., 2004). The Perceptions of Nurse Work Environment scale used a four‐point Likert scale (1 = strongly disagree to 4 = strongly agree), where higher mean composite scores indicate a favourable NPE, and scores were aggregated to the hospital level (Stone et al., 2007).

Nine studies used two different versions of the PES‐NWI. The 31‐item PES‐NWI was used in seven studies (Aiken et al., 2008, 2011; Flynn et al., 2012; Friese et al., 2008; Ma & Park, 2015; McHugh et al., 2016; Olds et al., 2017), and the 29‐item PES‐NWI was used in two studies (Cho et al., 2015; Fasolino & Snyder, 2012). The PES‐NWI consists of five subscales; nurse participation in hospital affairs; nursing foundations of quality care; nurse manager ability, leadership and support of nurses; staffing and resource adequacy; and collegial nurse‐physician relationships. For the studies which used PES‐NWI, six studies used all subscales to measure the NPE (Cho et al., 2015; Fasolino & Snyder, 2012; Flynn et al., 2012; Friese et al., 2008; Ma & Park, 2015; Olds et al., 2017), and two studies used four subscales (Aiken et al., 2011; McHugh et al., 2016). One study examined three subscales (nursing foundation for quality of care; nurse manager ability, leadership, and support; and collegial/physician relations; Aiken et al., 2008). All studies using the PES‐NWI used a four‐point Likert scale (1 = strongly disagree to 4 = strongly agree), and scores were aggregated to either the unit level (Flynn et al., 2012; Ma & Park, 2015) or hospital level (Aiken et al., 2008, 2011; Cho et al., 2015; Fasolino & Snyder, 2012; Friese et al., 2008; McHugh et al., 2016; Olds et al., 2017).

Several approaches were taken to determine whether the NPE was favourable. Three studies used the mean score of the composite PES‐NWI scale; where a higher mean composite score indicated a favourable NPE (Fasolino & Snyder, 2012; Flynn et al., 2012; Stone et al., 2007). This approach was used by Fasolino and Snyder (2012) (mean = 2.89, *SD* = 0.13) and Flynn et al. (2012) (mean = 2.94, *SD* = 0.34) using the PES‐NWI, and Stone et al. (2007) (mean = 2.9, *SD* = 0.25) using the Perceptions of Nurse Work Environment Scale. Two studies used the mean of the included PES‐NWI subscales with this composite hospital‐level score then standardized (mean = 0, *SD* = 1) (Aiken et al., 2011; Olds et al., 2017). Hence, a one‐unit change in the standardized PES‐NWI used in the model refers to one standard deviation improvement. On the other hand, one study measured the mean of the PES‐NWI subscales without standardization (mean = 2.95, *SD* = 0.24) (Ma & Park, 2015). This means that the coefficient describing the contribution of the PES‐NWI in the models in the Aiken et al. (2011) and Olds et al. (2017) studies needs to be interpreted differently to the Fasolino and Snyder (2012) and Flynn et al. (2012) studies, or the Ma and Park (2015) study. Lastly, the remaining studies counted the number of subscales above the median across hospitals (Aiken et al., 2008; Cho et al., 2015; McHugh et al., 2016) or above a threshold of 2.5 (Friese et al., 2008). These counts were then used to summarize a hospital's NPE categorically in different ways depending on the number of sub‐scales used; with three subscales 0 = poor, 1–2 = mixed, 3 = better (Aiken et al., 2008), with four subscales 0–1 = poor, 2–3 = mixed, 4 = good/better (Cho et al., 2015; McHugh et al., 2016), and with five subscales 0–1 = unfavourable, 2–3 = mixed, 4–5 = favourable (Friese et al., 2008).

### 4.3 Mortality

Mortality was measured in seven studies (Aiken et al., 2008, 2011; Cho et al., 2015; Friese et al., 2008; McHugh et al., 2016; Olds et al., 2017; Stone et al., 2007). Mortality was inconsistently defined across studies. Three studies used 30‐day mortality (where death occurred either in hospital or postdischarge within 30 days of admission) (Aiken et al., 2011; Cho et al., 2015; Olds et al., 2017). Three studies reported 30‐day inpatient mortality (Aiken et al., 2008; Friese et al., 2008; Stone et al., 2007), and one reported 30‐day inpatient survival (McHugh et al., 2016). All studies reported that a favourable NPE was associated with a reduction in the likelihood of mortality, but estimates of the contribution of the NPE to the reduction in mortality varied. Cho et al., (2015) estimated the odds of 30‐day mortality for surgical patients were 48% lower in hospitals with a favourable NPE (OR = 0.52, 95% CI: 0.31–0.88, *p* = .01) compared to those with a mixed/poor NPE. Aiken et al., (2011) and Olds et al. (2017) found that each one‐standard deviation increase in PES‐NWI reduced odds of 30‐day mortality by 7% (OR = 0.93, 95% CI: 0.90–0.96, *p* < .01) and 6% (OR = 0.94, 95% CI: 0.89–0.99, *p* < .035) respectively. Similarly, McHugh et al. (2016) identified that an unfavourable NPE led to a decrease in odds of survival to discharge by 16% (OR = 0.84, 95% CI: 0.71–0.99, *p* < .05) compared to those with mixed/good NPE. Friese et al., (2008) reported that an unfavourable NPE significantly increased odds of 30‐day mortality (OR = 1.37, 95% CI: 1.07–1.76, *p* < .05) compared to hospitals with a mixed/good NPE. In intensive care unit patients, Stone et al., (2007) found no association between mortality and the NPE (OR = 0.97, 95% CI: 0.90–1.02).

### 4.4 Medication error

Two of the included studies examined the relationship between the NPE and medication error, with one study assessing association using correlation (Fasolino & Snyder, 2012) and the other using hierarchical linear regression, accounting for clustering within hospitals (Flynn et al., 2012). In both studies, medication error was defined as number of medication errors per 1,000 patient bed days. Fasolino and Snyder (2012) identified that NPE had a weak positive association with medication error rate (*r* = .15, *p* < .01) instead of the expected inverse correlation. Flynn et al., (2012), found no association between the NPE and medication error rates (*β* = −0.26, *t* = −0.87, *p* < .388).

### 4.5 Pressure injury

The relationship between the NPE and pressure injury was examined in two studies (Ma & Park, 2015; Stone et al., 2007). In both studies, pressure injury was defined as the number of patients with any stage of pressure injury per 1,000 discharges, and the likelihood of a pressure injury developing was modelled. Ma and Park (2015) found a significant relationship between the NPE and development of pressure injury, where each one‐unit improvement in the composite NPE score led to reduced odds of developing a pressure injury by 29% (OR = 0.71, 95% CI: 0.55–0.91, *p* = .013). Stone et al., (2007) found no relationship between the NPE and the likelihood of developing a pressure injury (OR = 1.06, 95% CI: 0.83–1.37).

### 4.6 Hospital‐acquired infection

Hospital‐acquired infections were reported in only one of the ten studies (Stone et al., 2007). This study measured three types of hospital‐acquired infections within an intensive care setting; central line‐associated blood stream infection, catheter‐associated urinary tract infection and ventilator‐associated pneumonia. The association between the NPE and these was inconsistent. Each one‐unit improvement in the composite NPE was associated with a 39% reduction in the odds of developing a catheter‐associated urinary tract infection (OR = 0.61, 95% CI: 0.44–0.83), but a 19% higher odds for developing a central line‐associated blood stream infection (OR = 1.19, 95% CI: 1.05–1.36) (Stone et al., 2007). There was no significant association between a favourable NPE and development of ventilator‐associated pneumonia (OR = 1.03, 95% CI: 0.79–1.34) (Stone et al., 2007).

### 4.7 Patient fall

None of the included studies examined patient falls.

## 5 DISCUSSION

The aim of this study was to synthesize the literature on the relationship between the NPE and five selected nursing‐sensitive patient outcomes (mortality, medication error, pressure injury, hospital‐acquired infection and patient fall) from administrative data sources. Most studies reported that mortality was less likely in hospitals with a favourable NPE, but the considerable variance in the results suggests the magnitude of this relationship is unclear. This inconsistency may result in part from a discrepancy in the operational definitions of mortality used within included studies, differences in populations and settings or from inconsistencies in measurement/classification of favourable practice environments.

Surprisingly, none of the included studies examined the impact of the NPE on falls or falls resulting in patient harm. Many studies that use falls as a patient outcome examine nurses' perceptions of the numbers of falls as a proxy measure (Ausserhofer et al., 2013; Duffield et al., 2011; Swiger et al., 2018). However, studies that used nurse perceptions on the frequency and/or severity of adverse events were excluded from this review. The lack of empirical data examining the association between the NPE and falls should be addressed in future research because patient falls are a significant and frequent adverse event in hospitals (Manojlovich et al., 2016). Within the Australian healthcare system, 26,060 falls occur annually at a cost of $9.8 million each year to the Victorian and New South Wales states alone (Morello et al., 2015).

In this review, two studies examined medication errors. Fasolino and Snyder (2012) found that a favourable NPE led to higher rates of reported medication errors while Flynn et al. (2012) found no association between these variables. Reporting bias may influence the reliability of results on medication errors for two reasons. Firstly, under‐reporting of medication error is well documented in the literature (Pfeiffer et al., 2013) with the reasons including fear of consequences (Yung et al., 2016) and lack of knowledge about using the reporting system (Rutledge et al., 2018). Secondly, a favourable NPE may improve the safety culture and thus increase the reporting rate (Yoo & Kim, 2017). Therefore, increased rates of medication errors may result from increased reporting of medication errors rather than any change in the NPE. In addition, nurses make up only one part of the team that are responsible for medication errors. Nurses are involved in administration errors but prescribing and dispensing errors usually relate to other healthcare professionals (Doran, 2011). Neither study reported on the phase in which the error occurred (Fasolino & Snyder, 2012; Flynn et al., 2012). This means the errors may or may not have been due to the nurse incorrectly administering the medication and the NPE may not have had any association with medication error rates. In future research, measures of medication error at the administration stage would provide better insight into the relationship between the NPE and medication errors resulting from nursing care. Alternative sources of data, such as chart review or direct observation, could also be used to capture medication errors; which may be more reliable (Flynn et al., 2012).

Pressure injuries have been documented as an indicator of high quality nursing care (Rodgers et al., 2020a, 2020b; Stalpers et al., 2015). This review found mixed findings on whether the NPE was associated with pressure injury (Ma & Park, 2015; Stone et al., 2007). One study found that a favourable NPE reduced the odds of developing a pressure injury (Ma & Park, 2015). In contrast, Stone et al., (2007) found that the NPE was not associated with pressure injury development. These differences may relate to the different populations and research settings in each study. The study by Stone et al., (2007) was conducted among elderly people in intensive care units, where critically ill patients are at higher risk of developing skin breakdown (Coyer et al., 2017). Both studies reported that an increase in nursing hours per patient day reduced the rate of pressure injuries (Ma & Park, 2015; Stone et al., 2007). This indicates that nurse staffing and resource adequacy may be the key element of the NPE that influences the development of pressure injuries. Further studies are needed to strengthen the evidence on the relationship between the NPE and pressure injury and may include using risk adjustments to minimize confounding factors and control for nurse staffing.

The most common types of hospital‐acquired infections are central line‐associated blood stream infection, catheter‐associated urinary tract infection and ventilator‐associated pneumonia (Doran, 2011; Javadi Bashar, 2019). Mixed results were reported in the only included study that examined this outcome (Stone et al., 2007). Stone et al. (2007) conducted their study within intensive care settings in the USA where units are staffed by intensivists, respiratory therapists and Registered Nurses. It is possible that the mixed results relate to these different roles in intensive care settings in the USA and how care is organized. For instance, subclavian central‐line catheter insertion is usually performed by intensivists, but routine care of the central‐line is performed by Registered Nurses. Other factors such as overtime, staff education, training or experience may also be associated with the rate of hospital‐acquired infections (Stone et al., 2007). Further research is also required to explore hospital‐acquired infections in non‐critical care settings.

## 6 LIMITATIONS

Several limitations to this review need to be acknowledged. First, due to limiting this search to studies that included administrative data, only a small number of studies were identified. Second, all studies have used a cross‐sectional design, which does not support causal inference. Third, although a health service librarian assisted in the search, the search may not have identified all studies. Finally, because included studies used a variety of different conceptual definitions to measure outcomes and the heterogeneity in administrative data and populations, we were unable to perform a reliable meta‐analysis.

## 7 CONCLUSION

This review has identified that an improved NPE was associated with a reduction in mortality in seven studies. Due to variations in how mortality was defined and measured within included studies a meta‐analysis was not conducted. Other outcome measures (medication error, pressure injury and hospital‐acquired infection) only included small numbers of studies, and the association between the NPE and these patient outcomes was varied. No studies examined the association between the NPE and falls. More research needs to be done to understand the relationship between the NPE and these nursing‐sensitive patient outcomes in acute care hospital settings.

Improving the NPE is important for both nurses and patients. Nurses provide continuous care for hospitalized patients and this review demonstrates that a favourable NPE is associated with a reduction in mortality. Nurse Managers should actively promote improvements to the NPE to reduce patient mortality and potentially improve other patient outcomes. Supportive leadership and effective management practices should focus on ensuring appropriate evidence‐based nurse staffing, nurse participation in hospital decision‐making, effective collegial nurse–physician relationships and that the culture is focused on providing high quality patient care. Concepts such as nurse staffing and resource adequacy may be the key driver for improving some nursing‐sensitive patient outcomes such as pressure injuries (Shin et al., 2019). A favourable NPE may also improve the safety culture, which in‐turn leads to a non‐punitive response to errors and enables nurses to report and then learn from error (Chiang et al., 2017). Nursing leaders play an important role in improving the NPE and reducing preventable adverse events in hospital settings.

## CONFLICT OF INTEREST

The authors report no conflict of interest.

## AUTHOR CONTRIBUTIONS

TA, JS, LL: Substantial contributions to conception and design, or acquisition of data, or analysis and interpretation of data; Drafting of the manuscript or revising it critically for important intellectual content; Final approval of the version to be published and take responsibility for the content; Agreed to be accountable for all aspects of the work.

## PROSPERO REGISTRATION

The review protocol was registered (CRD42020143104) in the international prospective register of systematic reviews (PROSPERO) at the University of York Centre for Reviews and Dissemination on April 28, 2020.

## ETHICAL APPROVAL

This was a systematic review of previously published research and did not include human participants. It was therefore exempt from an ethics committee review.

## Supporting information

File S1

File S2

## Data Availability

The data used to support the findings of this study are available from the corresponding author upon reasonable request.
