# Supplementary material for: The relationship between the nursing practice environment and five nursing‐sensitive patient outcomes in acute care hospitals: A systematic review
Source: Nurs Open. 2021 Mar 4;8(5):2262–71. doi: 10.1002/nop2.828 (PMC8363353; doi:10.1002/nop2.828)
Supplement: Supplementary file 2 — File S2 [file NOP2-8-2262-s002.docx]

**Supplemental File 2**

Risk of Bias assessment for included studies

| **AXIS Critical Appraisal Questions (Downes, Brennan, Williams, & Dean, 2016)** | **Included Studies** | | | | | | | | | |
| --- | --- | --- | --- | --- | --- | --- | --- | --- | --- | --- |
|  | **Flynnn et al., (2012)** | **Aiken et al., (2008)** | **Olds et al., (2017)** | **Ma and Park, (2015)** | **Friese et al., (2008)** | **Cho et al., (2015)** | **Swiger et al., (2018)** | **Aiken et al., (2011)** | **Fasolino and Snyder, (2012)** | **McHugh et al., (2016)** |
| Were the aims/objectives of the study clear? | *✓* | *✓* | *✓* | *✓* | *✓* | *✓* | *✓* | *✓* | *✓* | *✓* |
| Was the study design appropriate for the stated aim(s)? | *✓* | *✓* | *✓* | *✓* | *✓* | *✓* | *✓* | *✓* | *✓* | *✓* |
| Was the sample size justified? | *X* | *✓* | *✓* | *X* | *✓* | *✓* | *X* | *✓* | *✓* | *✓* |
| Was the target/reference population clearly defined? (Is it clear who the research was about?) | *✓* | *✓* | *✓* | *✓* | *✓* | *✓* | *✓* | *✓* | *✓* | *✓* |
| Was the sample frame taken from an appropriate population base so that it closely represent the target/reference population under investigation | *✓* | *✓* | *✓* | *✓* | *✓* | *✓* | *X* | *✓* | *✓* | *✓* |
| Was the selection process likely to select subjects/participants that were representative of the target/reference population under investigation? | *✓* | *✓* | *✓* | *✓* | *✓* | *✓* | *X* | *✓* | *✓* | *✓* |
| Were measures undertaken to address and categorize non-responders? | *X* | *X* | *X* | *✓* | *X* | *X* | *✓* | *✓* | *U* | X |
| Were the risk factor and outcome variables measured appropriate to the aims of the study | *✓* | *✓* | *✓* | *X* | *✓* | *✓* | *✓* | *✓* | *✓* | *✓* |
| Were the risk factor and outcome variables measured correctly using instruments/measurements that had been trialed, piloted or published previously? | *✓* | *✓* | *✓* | *✓* | *✓* | *✓* | *✓* | *✓* | *✓* | *✓* |
| Is it clear what was used to determined statistical significance and/or precision estimates | *✓* | *✓* | *✓* | *✓* | *✓* | *✓* | *✓* | *✓* | *✓* | *✓* |
| Were the methods (including statistical methods) sufficiently described to enable them to be repeated? | *X* | *✓* | *✓* | *✓* | *✓* | *✓* | *✓* | *✓* | *✓* | *✓* |
| Were the basic data adequately described? | *X* | *✓* | *✓* | *✓* | *✓* | *✓* | *✓* | *✓* | *✓* | *✓* |
| Does the response rate raise concerns about non-response bias? | *X* | *X* | *X* | *X* | *X* | *X* | *X* | *✓* | *X* | *X* |
| If appropriate, was information about non-responders described? | *X* | *X* | *X* | *X* | *X* | *X* | *X* | *✓* | *X* | *X* |
| Were the results internally consistent? | *✓* | *✓* | *✓* | *✓* | *✓* | *✓* | *✓* | *✓* | *✓* | *✓* |
| Were the results presented for all the analyses described in the methods? | *X* | *✓* | *✓* | *✓* | *✓* | *✓* | *X* | *✓* | *X* | X |
| Were the authors' discussions and conclusions justified by the results? | *✓* | *✓* | *✓* | *✓* | *✓* | *✓* | *✓* | *✓* | *✓* | *✓* |
| Were the limitations of the study discussed? | *✓* | *✓* | *✓* | *✓* | *✓* | *✓* | *✓* | *✓* | *✓* | *✓* |
| Were there any funding sources or conflicts of interest that may affect the authors’ interpretation of the results? | *X* | *X* | *X* | *X* | *X* | *X* | *X* | *X* | *X* | *X* |
| Was ethical approval or consent of participants attained? | *✓* | *✓* | *✓* | *✓* | *✓* | *✓* | *✓* | *✓* | *✓* | *✓* |

*✓: Yes X: No U: Unknown*
